# Metformin anti-tumor effect via disruption of the MID1 translational regulator complex and AR downregulation in prostate cancer cells

**DOI:** 10.1186/1471-2407-14-52

**Published:** 2014-01-31

**Authors:** Ummuhan Demir, Andrea Koehler, Rainer Schneider, Susann Schweiger, Helmut Klocker

**Affiliations:** 1 Department of Urology, Innsbruck Medical University, 6020 Innsbruck, Austria; 2 Institute of Biochemistry, Center of Molecular Biosciences Innsbruck (CMBI), University of Innsbruck, 6020 Innsbruck, Austria; 3 Institute for Human Genetics, Medical School, University of Mainz, 55131 Mainz, Germany

**Keywords:** Metformin, Androgen receptor, MID1-α4/PP2A protein complex, AMPK, Translational regulation, CoIP

## Abstract

**Background:**

Metformin is an approved drug prescribed for diabetes. Its role as an anti-cancer agent has drawn significant attention because of its minimal side effects and low cost. However, its mechanism of anti-tumour action has not yet been fully clarified.

**Methods:**

The effect on cell growth was assessed by cell counting. Western blot was used for analysis of protein levels, Boyden chamber assays for analyses of cell migration and co-immunoprecipitation (CoIP) followed by western blot, PCR or qPCR for analysis of protein-protein and protein-mRNA interactions.

**Results:**

Metformin showed an anti-proliferative effect on a wide range of prostate cancer cells. It disrupted the AR translational MID1 regulator complex leading to release of the associated AR mRNA and subsequently to downregulation of AR protein in AR positive cell lines. Inhibition of AR positive and negative prostate cancer cells by metformin suggests involvement of additional targets. The inhibitory effect of metformin was mimicked by disruption of the MID1-α4/PP2A protein complex by siRNA knockdown of MID1 or α4 whereas AMPK activation was not required.

**Conclusions:**

Findings reported herein uncover a mechanism for the anti-tumor activity of metformin in prostate cancer, which is independent of its anti-diabetic effects. These data provide a rationale for the use of metformin in the treatment of hormone naïve and castration-resistant prostate cancer and suggest AR is an important indirect target of metformin.

## Background

Metformin is a commonly prescribed anti-diabetic drug. Epidemiological studies revealed a link between the use of metformin and a lower risk of several cancers, such as those of the breast, lung, colon and prostate [1,2]. On the other hand, a recent meta-analysis failed to find an influence of metformin on prostate cancer risk [3]. Despite these ambiguous data metformin inhibits many tumour cells in-vitro, including prostate cancer cells [4] and a number of clinical studies have been initiated to test the therapeutic efficacy of metformin in different cancer entities. Metformin targets several tumor-associated pathways [5,6], however, the mechanism of its anti-cancer activity is not yet fully understood.

In diabetic patients, metformin reduces hepatic glucose production by inhibiting gluconeogenesis. This effect is mainly achieved via inhibition of the mitochondrial respiratory chain I complex. This reduces the ATP/AMP ratio, which in turn activates AMPK and inhibits gene expression of gluconeogenesis enzymes and fructose-1, 6-biphosphatase activity thereby terminating gluconeogenesis. In addition, activation of AMPK also shifts cells from an anabolic to a catabolic state by inhibiting protein, glucose and lipid synthesis, and inducing glucose uptake by the glucose transporters GLUT1 and GLUT4 [7].

Whether the activation of AMPK by metformin underlies its anti-cancer effects remains a topic of debate. For example, AMPK inhibits mTOR, a key player in the protumorigenic PI3K-Akt-mTOR survival pathway [8], and also up-regulates the p53-p21 tumour suppressor axis [9]. However, studies in prostate cancer models have provided contradictory results. On the one hand inhibition of AMPK was reported to accelerate cell proliferation and promote malignant behaviour of tumour cells suggesting a tumour-suppressive activity [10]. On the other hand, increased AMPK activation via overexpression of its activator calmodulin kinase kinase was found in prostate cancer tumours, which stimulated growth and malignant properties of tumour cells [11,12].

Recently Kickstein et al. studied the action of metformin on tau phosphorylation in Alzheimer's disease [13]. The authors showed that metformin disturbs the assembly of the proteins midline-1 (MID1) and the regulatory (α4) and the catalytic subunits of protein phosphatase 2A (PP2A), which, together form a microtubule-associated ribonuclear protein complex [14]. Through the ubiquitin ligase activity of MID1 this complex acts as a negative regulator of protein phosphatase 2A (PP2A) by mediating its degradation [15]. Disruption of the MID1-α4/PP2A complex by metformin thus leads to increased PP2A activity. Due to the tumour-suppressive function of PP2A acting as an antagonist of protein kinases this may be relevant for the anti-tumour effects of metformin [16,17]. Loss of MID1 function due to mutations and subsequent overactivation of PP2A is found in Opitz G/BBB syndrome (OS) that is characterized by defects of midline organ development, e.g. heart, lip, palate, anus, and male urethra [15,18].

In addition to regulation of the PP2A phosphatase, the MID1-α4/PP2A complex also acts as a translational enhancer of complex-associated mRNAs [19,20]. Disruption of the complex by metformin is thought to affect translation of associated mRNAs, which bind via specific G-rich motifs and are transported to different cellular locations [14,19]. For example, huntingtin mRNA harbouring an extended CAG repeat is associated with and translationally-regulated by the MID1 complex [20].

The anti-tumour functions of PP2A and associated mRNAs suggest a regulatory role of the MID1 complex in cancer as well. In colorectal cancer a comparative study identified MID1 as one member of a 5-gene signature associated with lymph node involvement and overall survival [21]. With relevance to prostate cancer our previous investigations revealed an association of AR mRNA with the MID1 ribonuclear complex with AR mRNA via its trinucleotide repeat motifs and consequent upregulation of AR protein levels via this complex (unpublished results). Furthermore, we found overexpression of MID1 in prostate tumours, particularly those with a more aggressive phenotype.

These findings together with observations that metformin has beneficial effects in prostate cancer, and the data showing that metformin targets the MID1-α4/PP2A complex let us to hypothesize that metformin might interfere with AR protein synthesis via this complex and thus inhibit tumor properties of prostate cancer cells. We therefore investigated the action of metformin in a panel of benign and malignant prostate cell lines.

## Methods

### Reagents, chemicals and media

Compound-C (Sigma-Aldrich, St. Louis, MO, USA) was dissolved in DMSO, metformin and AICAR (both Sigma-Aldrich) were dissolved in water to prepare stock solutions. Cell culture media and supplements were obtained from PAA (Vienna, Austria), Pansorbin cells were from Calbiochem (Billerica, MA, USA). All reagents were from Sigma-Aldrich unless otherwise specified.

### Cell culture and cell counting

LNCaP, Du-145, VCaP and PC-3 cells were purchased from ATCC. DuCaP cells were a kind gift from Dr. Schalken, Nijmegen. The LNCaP-abl cell line, a model for castration-resistant prostate cancer, was established in our laboratory after long-term culturing in steroid-free medium [22]. The immortalized primary epithelial cell line RWPE1 was a generous gift from Dr. Watson (Dublin), EP156 cells were established by hTERT immortalization of primary epithelial prostate cells [23]. Media and culture conditions for cell lines are provided as Additional file 1: Supplementary methods. Cell numbers were determined using a cell counting system (Schaerf System, Reutlingen, Germany).

### Western blot analysis

Cells were lysed in RIPA buffer (50 mM Tris–HCl, pH 8.0, 150 mM NaCl, 0.5% Na-deoxycholate, 1% NP-40) supplemented with 1% phosphatase and 1% protease inhibitor cocktails, 5 mM NaF and 1 mM PMSF. Gel electrophoresis was performed according to standard protocols [24]. Antibodies and working dilutions for western blot: AR (1:100, Genetex, Irvine, CA, USA), GAPDH (1:100,000, Millipore, Billerica, MA, USA), AMPK and p-AMPK-Thr^172^ (1:1000, Cell Signalling, Danvers, MA, USA), MID1 (1:400, Sigma-Aldrich), α4 (1:500, Abcam, Cambridge, UK), N-flag (1:1000, Sigma-Aldrich), PP2A (1:1000, Millipore). Immunoblot bands were scanned and quantified using a scanning densitometer (Odyssey; Li-Cor Biosciences, Lincoln, NE, USA). The housekeeping protein GAPDH served as loading control.

### Cell transfections

Nanofectin (PAA) was used for transfection of cells with pCMV vectors containing full-length or Flag-tagged MID1 cDNA or empty vector (control) following the manufacturer’s recommendations. For siRNA transfection, α4-siRNAs were purchased from Dharmacon (Thermo-Fisher, Waltham, MA, USA), MID1-siRNA as reported previously [19] was purchased from GenXpress (Vienna, Austria). Nanofectin siRNA reagent (PAA) was used for siRNA transfections.

### Migration assay

After metformin treatment for 72 h, cells were seeded in 24-well BD cell culture inserts and metformin treatment was continued for a further 48 h. 20% FBS or 10% bovine serum (FBS) was used as chemo-attractants in the lower chamber for LNCaP or PC-3 cells, respectively. After 48 h, cells on the upper side of the membrane were removed by scraping with cotton swabs while cells on the lower side were fixed with methanol and stained with the nuclear stain DAPI. Cells that had migrated through the membrane were viewed with an immunofluorescence microscope (Carl Zeiss GMBH, Oberkochen, Germany) and quantified with TissueFAXs software (TissueGnostics, Vienna; Austria).

### Co-immunoprecipitation and analysis of associated proteins and mRNA

Cells were lysed in 100 mM NaCl, 20 mM Tris–HCl, 0.5 mM DTT, 10% glycerol and 0.1% NP-40 and pre-cleared with normal rabbit-serum-saturated pansorbin cells. After incubation with α4 antibody or rabbit control IgG (Santa Cruz, Dallas, TX, USA) overnight, the antigen-antibody complexes were immunoprecipitated with pansorbin cells. The pellets were washed four times with RIPA buffer. After boiling in SDS buffer, western blotting was performed with specific antibodies to visualize proteins interacting with α4. For RNA isolation from immunoprecipitates, poly(A) competitor RNA was added to pansorbin cells before pull-down and also to the last wash buffer. The pelleted pansorbin cells were washed four times with RIPA buffer supplemented with RNase inhibitor, and with metformin for the treated samples. Pellets were resuspended in RIPA buffer and Trizol® reagent, incubated at 65°C for 15 min and shaking, and total RNA was isolated following the protocol of the Directzol RNA extraction kit (Zymo Research, Irvine, CA, USA). RNA was reverse-transcribed to cDNA using the iScript select cDNA synthesis kit (Biorad, Hercules, CA, USA). An AR cDNA fragment containing the GAG repeat region was amplified using conventional PCR (GoTaq, Promega, Fitchburg, WI, USA), or AR mRNA was quantified by qPCR (ABI 7500 PCR System, Foster City, CA, USA). Primer and probe sequences and PCR conditions are provided as Additional file 1: Supplementary methods.

### Statistics

All numerical data are presented as mean ± SEM from at least three independent experiments. Values are shown relative to controls, which were set to 100%. Student’s t-test was used to compare groups. Statistically significant differences are denoted * p < 0.05, ** p < 0.01, *** p < 0.001.

## Results

### Metformin inhibits growth and reduces AR protein levels in prostate cancer cell lines

**Figure 1:**
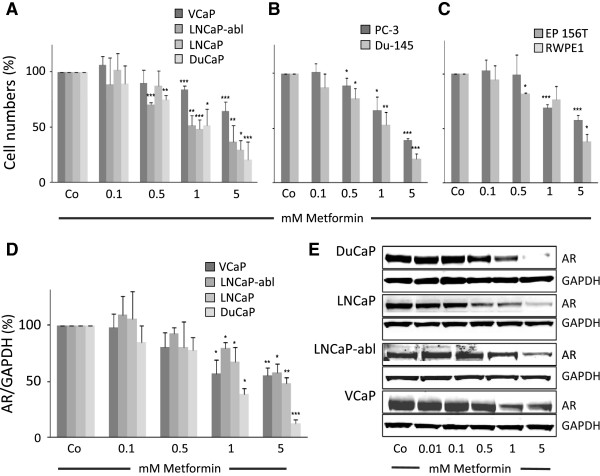
**The anti-diabetic drug metformin inhibits prostate cancer cell growth and reduces AR protein levels.** Prostate cancer and immortalized benign prostate epithelial cells were treated with increasing concentrations of metformin. After 96 h cell numbers were counted and AR levels determined by western blot. **(A)**, AR-positive cell lines, **(B)**, AR negative cell lines, and **(C)**, immortalized benign prostate epithelial cell lines. **(D, E)**, AR protein levels determined by quantification of western blot bands in AR positive cell lines. Each experiment was repeated at least 3 times. Representative western blot flouroscan images are shown in **(E)**. Statistical significant differences are indicated as *, p < 0.05; **, p <0.01 and ***, p < 0.001.

The anti-proliferative effect of metformin has been reported for LNCaP, C4-2, PC-3, and Du-145 prostate cancer cell lines. In our experimental setting, a wide range of prostate cell lines including AR-positive (LNCaP, VCaP, DuCaP, LNCaP-abl), AR-negative (PC-3 and Du-145), and benign epithelial cell lines (RWPE-1 and EP-156 T) were used to assess the effect of metformin (Figure 1A-C). Cell numbers decreased significantly after 96 h of treatment with increasing concentrations of metformin up to 5 mM. While metformin affected the proliferation of all cell lines tested, the benign prostate epithelial cells were the least sensitive and the androgen receptor positive cell lines DuCaP and LNCaP were the most sensitive ones. In the AR positive cell lines, AR protein levels decreased upon metformin treatment in a dose-dependent manner (Figure 1D, E). DuCaP cells, which showed the strongest anti-proliferative effect upon metformin treatment, also responded with the most significant AR downregulation. Of note, AR protein was also significantly downregulated in LNCaP-abl cells, which represent a castration-resistant prostate cancer phenotype.

### Metformin inhibits migration of prostate cancer cell lines

**Figure 2:**
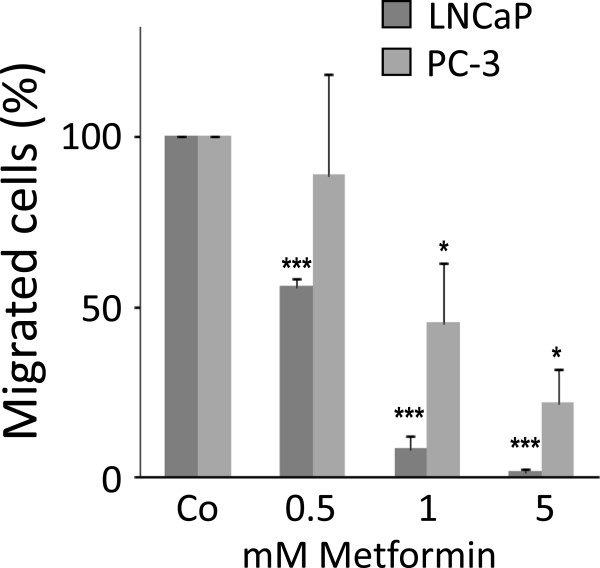
**Metformin inhibits cell migration.** After 72 hours of treatment with metformin, LNCaP and PC3 cells were seeded in Boyden chambers and the number of migrated cells was quantified 48 hours later. Each experiment was repeated at least 3 times. Statistical significant differences are indicated as *, p < 0.05; **, p <0.01 and ***, p < 0.001.

To determine whether metformin affects additional tumourigenic properties of cancer cells, we next investigated the effect of metformin on cell migration (Figure 2). Similar to proliferation, the inhibitory effect of metformin was again much more pronounced in the AR positive LNCaP than in the AR negative PC-3 cells.

### Activation of AMPK is not required for inhibition of prostate cancer cell proliferation by metformin

It is frequently presumed that the anti-proliferative effects of metformin are mediated via AMPK activation. Thus we first confirmed activation of AMPK in prostate cancer cells (Additional file 2: Figure S1). Indeed, in AR negative tumor cell lines Du145 and PC3 a significant increase of the active, phosporylated form of AMPK (P-AMPK) was detected by western blot at all time points up to 96 h of metformin treatment (Additional file 2: Figure S1A). Similarly, in AR positive cell lines LNCaP and DuCaP AMPK was activated after 24 h of treatment but abrogated after 96 h (Additional file 2: Figure S1B). This is to be expected since AMPK is activated in AR positive cell lines by the androgen-regulated calmodulin kinase kinase [12,25] and AR levels decrease in the course of metformin treatment.

**Figure 3:**
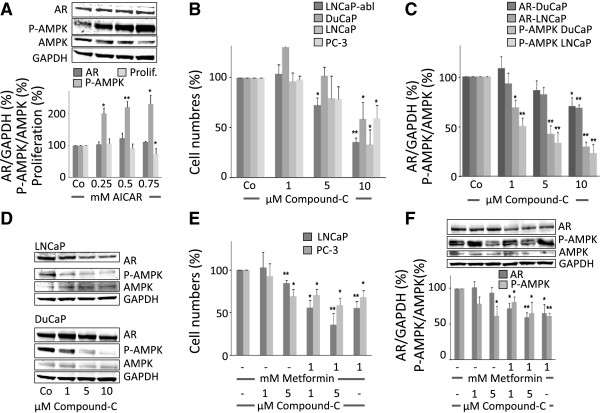
**AMPK activation is not required for metformin inhibition of prostate cancer cells.** Prostate cancer cells were treated with increasing concentrations of AICAR, an AMPK activator, Compound C, an AMPK inhibitor or a combination of AMPK inhibitor and metformin, respectively. LNCaP cells were treated with the AMPK activator AICAR. After 96 h AR levels were quantified by western blot and normalized to GAPDH. Activation of AMPK was verified by detection of phosphorylated AMPK by western blot (P-AMPK/AMPK ratio) **(A)**. Prostate cancer cells were treated with increasing concentrations of the AMPK inhibitor compound-C (0–10 μM) and cell numbers **(B)** and AR protein levels **(C)** in LNCaP and DuCaP cells were determined after 96 h. Inhibition of AMPK was verified by detection of phosporylated AMPK (P-AMPK/AMPK ratio) **(C)**). Representative fluoroscan images of LNCaP and DuCaP cell western blots are shown in **(D)**. Addition of 1 mM of metformin to the AMPK inhibitor compound-C resulted in enhanced inhibition of cell proliferation in LNCaP and PC3 cells **(E)** and further reduction of AR protein levels in LNCaP cells **(F)**. AR levels and P-AMPK/AMPK ratios in Figure 3 were quantified by western blot densitometry. The histograms show the data of at least three independent experiments, the fluoroscan image shows representative western blots. Statistical significant differences are indicated as *, p < 0.05; **, p <0.01 and ***, p < 0.001.

To test whether it is AMPK activation by metformin that mediates the inhibitory effect on prostate cancer cells we used another AMPK activator, the AMP mimetic AICAR. As expected, AMPK was activated as indicated by increased levels of the phosphorylated form (P-AMPK) (Figure 3A). In contrast to metformin however, despite strong AMPK activation by AICAR, this activator had a mild anti-proliferative effect only at the highest concentration used and AR protein levels remained unchanged (Figure 3A). These data indicate that AMPK activation is not required for inhibition of proliferation or down-regulation of AR protein level and another mechanism must be responsible for these metformin actions.

We next investigated whether AMPK inhibition could rescue metformin effects on cell proliferation and AR protein synthesis. The specific AMPK inhibitor compound-C alone exerted similar effects on cell proliferation and AR protein level as metformin, albeit less pronounced (Figure 3B-D). For example, at a concentration of 10 μM that almost completely prevented AMPK phosphorylation (10 μM, Figure 3C), compound-C resulted in an approximately 30% decrease in AR protein levels and cell number was decreased by approximately 50%. In combination, metformin and compound-C further inhibited cell growth and reduced AR protein level despite very low AMPK phosphorylation (Figure 3E, F). Collectively these data indicate that AMPK activation is dispensable for the inhibitiory actions of metformin on prostate cancer cells.

### Disruption of the MID1-α4/PP2A protein complex inhibits prostate cancer cell growth and decreases AR protein levels

**Figure 4:**
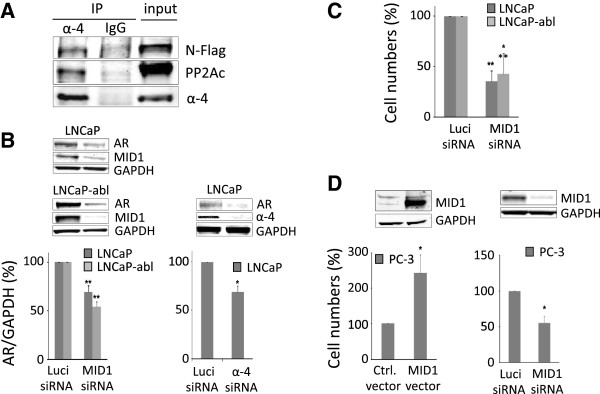
**Regulation of AR protein level and cell growth via the MID1-α4/PP2A transcriptional regulator complex.** MID1, α4 and PP2A form the core of a ribonuclear protein complex that enhances translation of associated mRNAs such as AR mRNA. Physical interaction of the complex components was confirmed in LNCaP cells overexpressing a flag-tagged MID1. MID1 and PP2A were co-precipitated in an α4 pull-down (α4); normal rabbit IgG was used as negative control (IgG). A representative western blot of two independent experiments is shown in **(A)**. Disruption of the complex by MID1 or α4 protein knockdown mimics the effect of metformin. MID1 knockdown in LNCaP and LNCaP-abl cells or α4 in LNCaP cells resulted in a reduction of AR protein levels **(B)** and the inhibition of cell growth **(C)**. In the AR-negative PC3 cells, MID1 overexpression enhanced, whereas MID1 knockdown inhibited cell growth **(D)**. Successful knockdown or overexpression, respectively, was verified by western blot; inserts show representative fluoroscans **(B, D)**. Luciferase (Luci) siRNA was used as negative control for siRNA transfections, empty vector for overexpression control. Cell numbers were determined after 10 days. Statistical significant differences are indicated as *, p < 0.05; **, p = <0.01 and ***, p < 0.001.

Metformin targets the MID1-α4/PP2A translational regulator complex and was previously shown to dissociate the complex and release MID1 and α4 proteins from PP2A [13]. After exclusion of AMPK as the responsible target, we hypothesized that interference with this protein complex is responsible for the effects of metformin on prostate cancer cells. To further elucidate this mechanism we used α4 antibody pull-down in LNCaP cells overexpressing flag-tagged MID1 to confirm the physical association of MID1, α4 and PP2A in these cells (Figure 4A). In a next step, disruption of the MID1 protein complex by siRNA knockdown of either MID1 or α4 was carried out. MID1 significantly reduced AR protein levels in LNCaP and LNCaP-abl cells (Figure 4B). The same effect was achieved with α4 knockdown as shown for LNCaP cells (Figure 4B). Disruption of the complex by siRNA knockdown resulted in decreased proliferation of the AR positive cell lines similarly to what we observed with metformin (Figure 4C). Interestingly, MID1 knockdown also exerted an inhibitory effect on AR negative PC-3 cells whereas overexpression increased cell numbers (Figure 4D) indicating that AR protein synthesis is not the single and only target of metformin. The opposite effect on AR protein levels was observed upon MID1 overexpression in LNCaP cells (Additional file 3: Figure S2A), however AR negativity of PC3 cells remained unchanged upon MID1 overexpression (Additional file 3: Figure S2B).

### Metformin disrupts the association of AR mRNA with the MID1 complex

**Figure 5:**
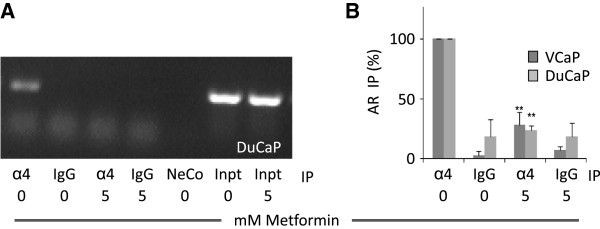
**Metformin disrupts the MID1-α4/PP2A complex and releases associated AR mRNA.** DuCaP or VCaP prostate cancer cells were treated with 5 mM of metformin or vehicle control for 24 h. Afterwards the MID1-α4/PP2A complex was immunoprecipitated using an α4 specific antibody. Normal rabbit IgG was used as negative control. Complex-associated RNA was isolated and transcribed to cDNA. Using PCR and real-time PCR amplification of an AR cDNA fragment containing the CAG-repeat region **(A)** or a fragment of the AR hormone-binding region **(B)**, respectively, were amplified. The agarose gel image **(A)** shows a representative PCR result of 3 independent experiments with DuCaP cells, the histogram **(B)** shows the relative real-time PCR AR fragment levels standardized to the input amount and normalized to the control for 3 independent experiments for DuCaP and VCaP cells. NeCo, negative control; Inpt, input. PCR primer and fragment information is provided in the supplementary data. Statistical significant differences are indicated as *, p < 0.05; **, p <0.01 and ***, p < 0.001.

The MID1-α4/PP2A complex binds mRNA containing purine-rich sequences including so called MIDAS motifs and trinucleotide repeats [19,20]. AR mRNA is one of the bound mRNAs. Thus, we therefore proposed that metformin may cause disassociation of the AR mRNA from the complex. To test this notion we immunoprecipitated the complex from control or metformin treated DuCaP and VCaP prostate cancer cells using an α4 antibody. AR mRNA was detected in α4-IP samples but was absent or strongly reduced in samples pre-treated with 5 mM metformin (Figure 5A, B) as shown by PCR amplification of a cDNA fragment containing the AR CAG region (Figure 5A) or by qPCR of an AR cDNA fragment of the hormone binding domain (Figure 5B). On the other hand metformin treatment did not result in a change of the overall protein level of the catalytic subunit of PP2A under the conditions used in our experiments (Additional file 4: Figure S3). Taken together these data confirm that the MID1-α4/PP2A complex with its associated mRNAs is a target for metformin and provides a mechanism for AR protein downregulation by metformin.

## Discussion

The anti-tumour effect of metformin has been observed in different types of cancers but a clear mechanism of action remained elusive. Several clinical trials are currently being performed to assess the effect of metformin alone or in combination with different drugs in various types of cancer including prostate cancer [26]; ( http://www.clinicaltrials.gov, https://www.clinicaltrialsregister.eu). A better knowledge of the cellular target(s) and the molecular mechanism of metformin action could support patient selection and optimize treatment regimens in order to achieve optimal therapeutic efficacy.

Metformin has a well-documented effect on the translation of mRNAs. However, its effects do not globally inhibit translation such as expected when cells attempt to spare energy, rather, its inhibitory effects are restricted to a specific pool of mRNAs [27]. In our previous investigations we established that the MID1-α4/PP2A ribonuclear protein complex regulates AR protein levels in a post-transcriptional manner (unpublished results). The results presented herein establish a link between the effect of metformin and AR via this translational regulator complex. Kickstein et al. [13] demonstrated disruption of the MID1-α4/PP2A complex and release of MID1 and α4 proteins from anchored PP2A by metformin in an *in-vitro* reconstitution model. In agreement with this mechanism of action, our data show that metformin promotes the release of AR mRNA associated with the complex resulting in AR protein downregulation and subsequent growth inhibition of prostate cancer cells. Accordingly, disruption of the complex by silencing either MID1 or α4 yielded the same outcome as treatment with metformin. Of the prostate cancer cells tested, AR positive cell lines were most sensitive to the inhibitory effects of metformin supporting the conclusion that metformin mediates this action at least in part via reduction of AR protein levels. In agreement with our findings Colquhoun et al. reported inhibition of colony formation in AR positive LNCaP cells at much lower metformin concentrations than in AR negative PC-3 and Du-145 cells and enhancement of the antiproliferative effects of the antiandrogen bicalutamide [28]. Consistent with data of Ben Sahra et al. we also observed that benign cell lines were least sensitive to metformin [4]. However, AR negative cell lines were also inhibited by metformin, suggesting additional targets in addition to the AR. In this respect, a likely candidate is the PTEN-Akt pathway, which supports proliferation, survival and migration of prostate cancer cells. Moreover, the PTEN-Akt pathway is often overactivated in prostate cancer via loss or inactivation of the tumour suppressor PTEN [29,30]. Disruption of the MID1-α4/PP2A complex targets the PTEN-Akt pathway by interfering with the translation of the Akt-kinase PDPK-1 and enhancing the activity of the protein kinase antagonist PP2A [19]. Importantly in terms of prostate cancer treatment LNCaP-abl cells, which represent a model of castration resistant prostate cancer with gain of AR function [22], were also highly sensitive to metformin treatment. This suggests efficacy of metformin in castration resistant prostate cancer and recommends in particular a combination of metformin with other drugs in late stage disease. In support of the hypothesis that metformin mediates its actions at least in part by modulating AR protein levels, metformin was found to reduce serum androgen levels and endometrial AR levels in polycystic ovarian syndrome (PCOS), a disease characterized by elevated action of androgen and/or AR [7,31].

A concern expressed about the use of metformin in cancer patients is its unclear effect on glucose levels in non-diabetic patients. It has been suggested that metformin reduces blood glucose levels only in diabetics, but not so in non-diabetics [5]. This is consistent with the preliminary results of clinical trials, which show that metformin does not induce hypoglycemia [32]. Our data suggest that metformin’s anti-proliferative effect on prostate cancer cells does not require AMPK activation, which, as a metabolic sensor, is the main effector molecule of metformin on metabolism and inhibition of gluconeogenesis. The AMPK activator AICAR showed no significant effect on proliferation or AR protein levels, when used at concentrations that exerted AMPK activation similar to metformin. Only at the highest inhibitor concentration a mild inhibitory effect on cell proliferation was noticed. This might be a sign of unspecific toxicity or might indicate an additional role of AMPK. In the contrary to the activator AICAR, the AMPK inhibitor compound C decreased AR levels, albeit less than metformin, attenuated proliferation and exerted a synergistic inhibitory effect together with metformin. This agrees with recent investigations that found AMPK to be over-activated via CAM kinase kinase in prostate tumours and that it promotes tumour progression and development of castration resistance [11,12]. Taken together these data provide evidence that activation of AMPK is not a determinant for the inhibitory effects of metformin on prostate cancer cells.

The migration potential of cancer cells is essential for the development of metastases. Metformin inhibited the migration of AR-positive as well as AR-negative prostate cancer cells. Again the effect was more pronounced in the AR-positive cells. It was recently reported that activation of PP2A via inhibition of MID1 reduced the migration of neural crest cells [33]. Metformin might mediate a similar effect in AR negative and positive prostate cancer cells in addition to its ability to downregulate AR. Furthermore, mesenchymal-to-epithelial transition (EMT) stimulated by TGF-β and its interplay with AR signaling is important for prostate cancer cell migration [34,35]. Metformin was found to inhibit EMT by interfering with TGF-β regulation in renal and in breast cancer cells [36,37] and by modulating AR translation as shown herein and other EMT effectors such as MMP14 [19].

## Conclusions

In conclusion the results of our study support the use of metformin for treatment of all stages of prostate cancer. The standard treatment for advanced prostate cancer is androgen deprivation therapy. It is initially effective in the majority of tumours but its long-term use is associated with side effects such as cardiovascular problems, metabolic disease, diabetes mellitus, and development of therapy resistance [38]. A combination of metformin with androgen deprivation might be a promising combination to improve efficacy and relieve side effects. Upregulation of AR via enhanced activity of the MID1 translational regulator complex could be abrogated by metformin and improve androgen deprivation therapy. Our data confirm that the MID1-α4/PP2A ribonuclear protein complex is a target for the anti-tumourigenic effects of metformin. Metformin disrupts the MID1 protein complex and reduces AR protein levels in prostate cancer cells identifying AR as an indirect metformin target. A better understanding of the mechanism of action will support the setup and interpretation of clinical studies and help to optimize treatment efficacy and minimize side effects.

## Abbreviations

CoIP: Co-immonoprecipitation; EMT: Epithelial to mesenchymal transition; FBS: Fetal bovine serum; AICAR: 5-Aminoimidazole-4-carboxamide 1-β-D-ribofuranoside.

## Competing interests

The authors declare that they have no competing interests.

## Authors’ contributions

UD designed and carried out the experiments analyzed the data, performed statistical analysis and drafted the manuscript. AK provided methodological help. RS, SS and HK conceived and designed the study and participated in the drafting of the manuscript. HK coordinated the study and finalized the manuscript. All authors read and approved the final manuscript.

## Pre-publication history

The pre-publication history for this paper can be accessed here:

http://www.biomedcentral.com/1471-2407/14/52/prepub

## Supplementary Material

Additional file 1 Supplementary methods. Click here for file

Additional file 2: Figure S1 Activation of AMP kinase by metformin. AR-negative **(A)** and -positive **(B)** prostate cancer cell lines were treated with increasing concentrations of metformin for 24 or 96 hours and AR, AMPK and P-AMPK levels were detected by western blot. In the AR negative cell lines PC3 and DU145 both short (24 h) and long (96 h) exposure of cells to metformin resulted in a dose dependent activation of AMPK **(A)**. In the AR positive cell lines DuCaP and LNCaP metformin treatment for 24 h increased P-AMPK similarly, albeit less steeply than in AR-negative cell lines due to their higher basal levels of P-AMPK. After prolonged (96 h) treatment, AMPK phosphorylation was abrogated, in LNCaP cells the P-AMPK/AMPK ratio even decreased compared to untreated cells. Representative western blot fluoroscan images are shown in **A** and **B**. The histograms at the bottom represent means and standard deviations of densitometric quantification of western blots of three independent experiments. Statistical significant differences are as *, p < 0.05; **, p = <0.01 and ***, p < 0.001. Click here for file

Additional file 3: Figure S2 AR is up-regulated upon MID1 overexpression. LNCaP or PC3 cells were transfected with a tagged-MID1 cDNA expression plasmid or empty expression vector as a control. After 72 h cells were harvested and overexpression was verified by western blot. Proteins as indicated were determined by western blot. The histogram shows the densitometric analysis of three independent experiments with LNCaP cells. The western blots show fluoroscan images of representative experiments. In LNCaP cells MID1 overexpression resulted in AR upregulation **(A)**, however, the AR-negative status of PC3 cells was not changed by MID1 overexpression **(B)**. Click here for file

Additional file 4: Figure S3 Metformin treatment does not change PP2A protein level in prostate cancer cells. AR-positive prostate cancer cell lines DuCaP and LNCaP were treated with increasing concentrations of metformin for 24 h or 96 h, respectively. Cells were harvested and PP2A was detected by western blot. The fluoroscan images show representative western blots of PP2A and the house-keeping protein GAPDH. Click here for file
