# Supplementary material for: Metformin anti-tumor effect via disruption of the MID1 translational regulator complex and AR downregulation in prostate cancer cells
Source: BMC Cancer. 2014 Jan 31;14:52. doi: 10.1186/1471-2407-14-52 (PMC3929757; doi:10.1186/1471-2407-14-52)
Supplement: Additional file 1 — Supplementary methods. [file 1471-2407-14-52-S1.docx]

**Additional file 1: Supplementary methods.**

**Cell Culture.** Prostate tumor and immortalized benign epithelial prostate cells were routinely cultured at 37°C in a humidified atmosphere of 5% CO2 in air using the media:

humidified atmosphere of 5 % CO2 in air using the following media:

| Cell line | PC-3 | Du-145 | LNCaP | LNCaP-abl | DuCaP | VCaP | RWPE1 | EP156T |
| --- | --- | --- | --- | --- | --- | --- | --- | --- |
| Basal Medium | RPMI | RPMI | DMEM | RPMI | RPMI | DMEM | K-SFM | MCDB153 |
| FBS | 10% | 10% | 10% | 10%* | 10% | 10% |  | 1% |
| 100x glutamax solution | 1% | 1% | 1% | 1% | 1% | 2% | 1% | 1% |
| 100x pen/ strep solution | 1% | 1% | 1% | 1% | 1% | 1% | 1% | 1% |
| Na-pyruvate |  |  | 1 mM | 1 mM |  |  |  |  |
| D-Glucose |  |  | 2.3 g/L | 2.3 g/L |  | 2.3 g/L |  |  |
| HEPES pH=7.2 |  |  | 10 mM |  |  |  |  |  |
| BPE |  |  |  |  |  |  | 0.05mg/ml | 50ng/ml |
| EGF |  |  |  |  |  |  | 5 ng/ml | 5 mg/ml |
| R1881 |  |  |  |  |  |  |  | 10 nM |

* Char-coal stripped, steroid-free FCS

**Androgen receptor PCR primers and Probes:** For amplification of a cDNA fragment of 295 bp in length containing the CAG triplet repeat (AR mRNA transcript variant 1, bases 1175 – 1470) the forward primer: 5-'AGGAGCTTTCCAGAATCTGTT-3' and the reverse primer 5-GCTGTGAAGGTTGCTGTTCCTC-3’ were used. PCR amplification was performed with initial denaturation for 2 min followed by 5 cycles with for 30s at 95°C, annealing for 1.5 min at 56°C and synthesis for 40s at 72°C and 27 cycles with denaturation for 30s at 95°C, annealing for 1min at 55°C and synthesis for 40s at 72°C followed by a final synthesis step of 5min at 72°C. The PCR product was visualized in a 2% agarose gel using SybrGreen staining.

AR mRNA quantification by qPCR followed the Taqman technique. Primers 5`AGGATGCTCTACTTCG CCCC-3` (forward) and 5`-ACTGGCTGT ACATCCGGGAC-3` (reverse) and the Taqman probe FAM-5`-TGGTTTTCAATGAGTACCGCATGCACA-3`-TAMRA were used to amplify an 72 bp AR cDNA fragment of the ligand-binding domain (AR mRNA transcript variant 1 bases 3396-4368) applying the standard protocol of the ABI 7500 real-time PCR system.
