# Supplementary material for: Metformin anti-tumor effect via disruption of the MID1 translational regulator complex and AR downregulation in prostate cancer cells
Source: BMC Cancer. 2014 Jan 31;14:52. doi: 10.1186/1471-2407-14-52 (PMC3929757; doi:10.1186/1471-2407-14-52)
Supplement: Additional file 2: Figure S1 — Activation of AMP kinase by metformin. AR-negative (A) and -positive (B) prostate cancer cell lines were treated with increasing concentrations of metformin for 24 or 96 hours and AR, AMPK and P-AMPK levels were detected by western blot. In the AR negative cell lines PC3 and DU145 both short (24 h) and long (96 h) exposure of cells to metformin resulted in a dose dependent activation of AMPK (A). In the AR positive cell lines DuCaP and LNCaP metformin treatment for 24 h increased P-AMPK similarly, albeit less steeply than in AR-negative cell lines due to their higher basal levels of P-AMPK. After prolonged (96 h) treatment, AMPK phosphorylation was abrogated, in LNCaP cells the P-AMPK/AMPK ratio even decreased compared to untreated cells. Representative western blot fluoroscan images are shown in A and B. The histograms at the bottom represent means and standard deviations of densitometric quantification of western blots of three independent experiments. Statistical significant differences are as *, p < 0.05; **, p = <0.01 and ***, p < 0.001. [file 1471-2407-14-52-S2.pdf]

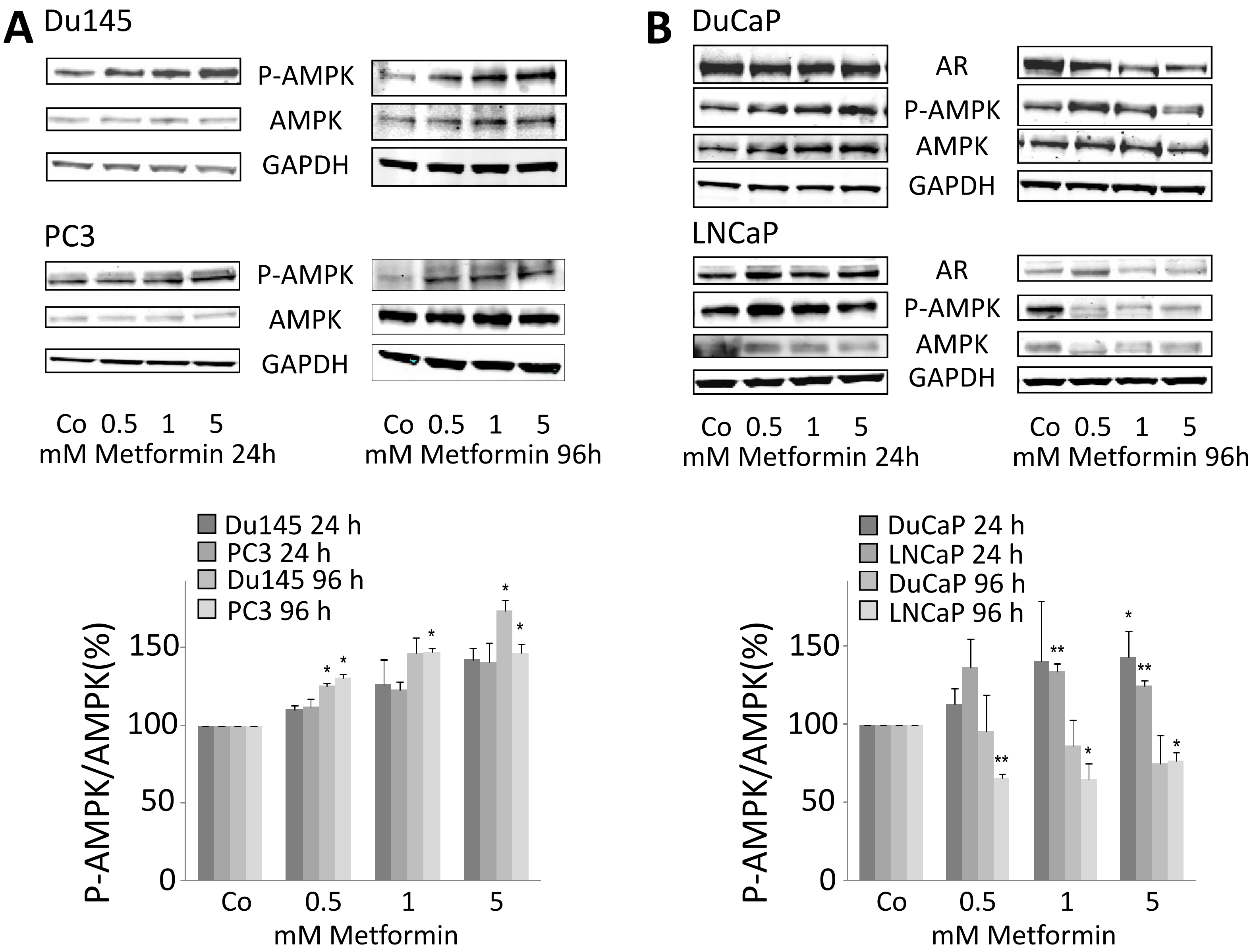

Supplementary Fig. S1
