# Supplementary figures and images for: Metformin anti-tumor effect via disruption of the MID1 translational regulator complex and AR downregulation in prostate cancer cells
Source: BMC Cancer. 2014 Jan 31;14:52. doi: 10.1186/1471-2407-14-52 (PMC3929757; doi:10.1186/1471-2407-14-52)

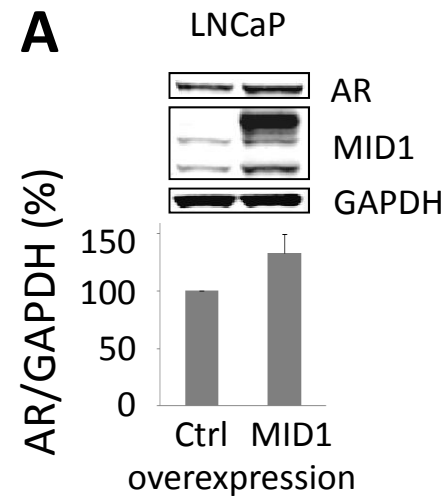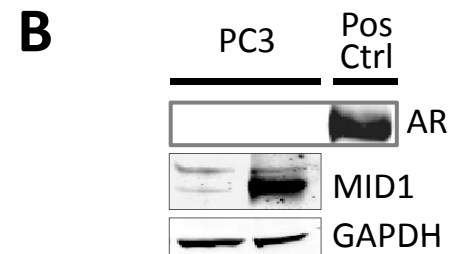

Supplementary Fig. S2

Supplement: Additional file 3: Figure S2 — AR is up-regulated upon MID1 overexpression. LNCaP or PC3 cells were transfected with a tagged-MID1 cDNA expression plasmid or empty expression vector as a control. After 72 h cells were harvested and overexpression was verified by western blot. Proteins as indicated were determined by western blot. The histogram shows the densitometric analysis of three independent experiments with LNCaP cells. The western blots show fluoroscan images of representative experiments. In LNCaP cells MID1 overexpression resulted in AR upregulation (A), however, the AR-negative status of PC3 cells was not changed by MID1 overexpression (B). [file 1471-2407-14-52-S3.pdf]

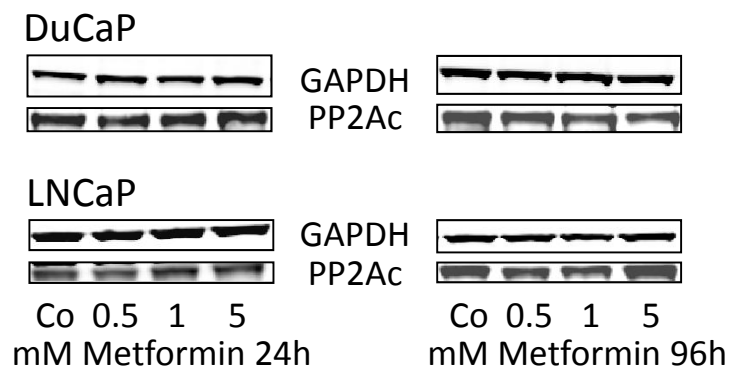

Supplementary Fig. S3

Supplement: Additional file 4: Figure S3 — Metformin treatment does not change PP2A protein level in prostate cancer cells. AR-positive prostate cancer cell lines DuCaP and LNCaP were treated with increasing concentrations of metformin for 24 h or 96 h, respectively. Cells were harvested and PP2A was detected by western blot. The fluoroscan images show representative western blots of PP2A and the house-keeping protein GAPDH. [file 1471-2407-14-52-S4.pdf]
